# Post-Irradiation Behavior of Colored PVA-Based Films Containing Ag Nanoparticles as Radiation Detectors/Exposure Indicators

**DOI:** 10.3390/gels10050290

**Published:** 2024-04-24

**Authors:** Linas Kudrevicius, Evelina Jaselskė, Gabrielius Stankus, Shirin Arslonova, Diana Adliene

**Affiliations:** 1 Physics Department, Kaunas University of Technology, 51368 Kaunas, Lithuania; gabrielius.stankus@ktu.edu; 2 Neurosurgery Department, Lithuanian University of Health Sciences, 44307 Kaunas, Lithuania; 3 Tashkent City Branch of Republican Specialized Scientific-Practical Medical Centre of Oncology and Radiology, Boguston Str. 1, Tashkent P.O. Box 100070, Uzbekistan

**Keywords:** radiation detection, polymer gel, radiation exposure indicators, colored films, film dosimetry

## Abstract

Ionizing radiation covers a broad spectrum of applications. Since radioactive/radiation pollution is directly related to radiation risk, radiation levels should be strictly controlled. Different detection methods can be applied for radiation registration and monitoring. In this paper, radiation-induced variations in the optical properties of silver-enriched PVA-based hydrogel films with and without azo dye (Toluidine blue O, TBO, and Methyl red, MR) additives were investigated, and the feasibility of these free-standing films to serve as radiation detectors/exposure indicators was assessed. AgNO_3_ admixed with PVA gel was used as a source for the radiation-induced synthesis of silver nanoparticles (AgNPs) in irradiated gel films. Three types of sensors were prepared: silver-enriched PVA films containing a small amount of glycerol (AgPVAGly); silver-enriched PVA films with toluidine blue adducts (AgPVAGlyTBO); and silver-enriched PVA films with methyl red additives (AgPVAGlyMR). The selection of TBO and MR was based on their sensitivity to irradiation. The irradiation of the samples was performed in TrueBeam2.1 (VARIAN) using 6 MeV photons. Different doses up to 10 Gy were delivered to the films. The sensitivity of the films was assessed by analyzing the characteristic UV-Vis absorbance peaks on the same day as irradiation and 7, 30, 45, 90, and 180 days after irradiation. It was found that the addition of azo dyes led to an enhanced radiation sensitivity of the AgNPs containing films (0.6 Gy^−1^ for AgPVAGlyTBO and 0.4 Gy^−1^ for AgPVAGlyMR) irradiated with <2 Gy doses, indicating their applicability as low-dose exposure indicators. The irradiated films were less sensitive to higher doses. Almost no dose fading was detected between the 7th and 45th day after irradiation. Based on the obtained results, competing AgNP formation and color-bleaching effects in the AgPVAGly films with dye additives are discussed.

## 1. Introduction

Ionizing radiation is widely used and well-monitored in different fields of medicine and industry, but the detection of ambient radiation exposure, especially in connection with unexpected radiation releases, remains one of the prevailing problems due to strict requirements for the accuracy, precision, and resolution of dose assessment methods [1]. Radiation exposure is still overestimated during aircraft flights at high altitudes [2] or in the space industry. People taking part in these activities are presumed to have a higher risk of developing cancer due to the increase in radiation levels at increased elevations [3]. The radiation level and exposure during flight can be calculated using computer algorithms [4]. However, new studies have shown that the calculated ambient dose H* for passengers and crew members in the aircraft industry lead to an overestimated effective dose *E* by up to 30–35% [5,6]. Different detection systems and electronic equipment [7], like commercially available 2D gafchromic films [8], chemical dosimeters, and especially 3D gel-based dosimeters, are suitable for radiation detection/radiation exposure assessment [9]. They can also be applied in stereotactic radiotherapy and radiosurgery for high-dose registration [10,11,12,13,14,15,16]. To overcome the problem with a single use of 3D polymer gel dosimeters, it has been suggested to use PVA-NaI gel dosimeters instead, which can be reset by heating at 60 °C and later re-used again with about a 4% variation in the dosimetry parameters [17]. Also, Tetrazolium Gellan dosimeters can be used, since they have a noticeable dose rate dependency at the low volumes used in clinical settings [18]. TBO-Pluronic F-127-based gel dosimeters have been proposed in various studies for 2D or 3D ultraviolet (UV) radiation detection [19,20,21]. There are a variety of different types of radiochromic dosimeters that change color upon irradiation in the kGy dose range. However, in medical, especially in diagnostic, applications, this dose range is too large [22]. For low-dose gamma radiation detection, thin TeO_2_ films with a sensitivity of 1.2–37.0 nA/cm^2^/µGy and a detection range of 0.22–2.16 mGy have been proposed and investigated in the literature [23]. Another type of low-dose detectors that have been studied are Li_2_B_4_O_7_ thermally stimulated luminescence single crystals doped with 1% Ag. This type of detector fades up to 15% in the dark over 20 days in the irradiation range of 0.2–100 Gy [24]. However, it is important to highlight that there is a lack of dosimeters that have been evaluated in long-term stability studies [25]. Thus, it is of critical importance to develop and investigate dosimeters sensitive in a low dose range with a high stability over a substantial period.

Multilayer thin films of ZnO/Ag/ZnO irradiated with 1 Gy and 4 Gy doses and evaluated for 45 days were also investigated. The optical fading of the stored dose-related signal showed an acceptable linearity [26]. It has been observed that Ag-doped ZnO TLD films have a 1.8 times higher sensitivity compared to TLD 100 chips. However, the signal of former films, irradiated with 1 Gy and 4 Gy doses, fades by 8% and 20%, respectively, over the first hour. In direct sunlight, the stored information in the samples fades up to 70% over 6 h [27]. Ag-doped phosphate glass with lithium fluoride (LiF) is a novel 2D disk-type thin film dosimeter based on radiophotoluminescence (RPL) and photoluminescence (PL) phenomena, which can detect exposure in the diagnostic range [28]. CIGS solar cells can also be adopted for real-time therapeutic 6 MeV radiation detection in the range of 0–12 Gy [29]. In addition, halide perovskite semiconductors have also been proposed for ionizing radiation detection due to their minimal cost, ease of manufacturing, and many other parameters that make the handling of such detectors convenient [30]. There are also many published articles and ongoing studies regarding polymer-based gel dosimeters. For instance, in PAGAT (Poly Acrylamide Gelatin gel fabricated at atmospheric conditions) gels, when enriched with silver nanoparticles, the dose–response increases by up to 11.82% in a dose range up to 12 Gy [31,32]. Another additive that influences the sensitivity of dosimeters is isopropanol radicals (CH_3_)_2_CHOH). This effect is due to the ability of the alcohol radicals to further reduce Ag^+^ ions to Ag^0^ by up to 14% (this was observed for a PVA dosimeter, enriched with AgNPs, especially in the dose range of 5–10 Gy). Of course, this effect depends on the concentration of the isopropanol (0–30%) [33]. The effect of gold nanoparticles (AuNPs) in a PVA matrix has also been investigated, but the irradiation dose range was much larger (50–300 kGy). However, with AuNPs, it is possible to detect an LSRP peak at 540 nm using UV-Vis spectroscopy. Thus, this could be more suitable in industrial or material processing fields [34]. Another type of dosimeter for radiation detection via luminescence is layer-by-layer films, containing NaCl with AgNPs on glass/aluminum substrates [35]. An LSRP peak at a 450 nm wavelength was observed for AgNPs in 8% gelatin when the samples were irradiated up to 100 Gy [36]. The application of easy-to-handle 2D-type dosimeters in the form of reliable portable colorimeters with a low variability (CV < 3%) is also possible [37]. Cheap radiochromic dosimeters and different chemical composition dosimeters based on PVA and AgNPs were also investigated [38]. One additive that was interesting to investigate was sodium citrate (C_6_H_5_O_7_Na_3_), which was used in different concentrations to promote the enhanced generation of AgNPs in nMAg gels, as nanoparticle synthesis was observed at low doses [39]. Another study suggested adding ethanol (C_2_H_5_OH) to increase the PVA-Ag film sensitivity in the dose range of up to 4 Gy [40]. It is important to emphasize that, for PVA-Ag dosimeters, it is suggested to keep the pH level of the solution at about 5 (at least in the dose range between 0 and 100 Gy). This induces a blue shift of LSPR to shorter wavelengths [41]. In another study, a dosimetric film system was supplemented with glycerol as an additive to increase the flexibility of the films. It was also indicated that radicals of this polyhydroxy alcohol were also capable of reducing Ag^+^. In the same article, the irradiation of polyacrylamide-based hydrogel dosimeters containing AgNO_3_ with ^60^Co gamma rays in the dose range of 0–100 Gy was discussed. These hydrogels were stored for up to 20 days in a dark environment at 6 °C and 23 °C. It was found that the relative response was almost completely stable over 15 days, but at room temperature, the response increased quite drastically. Thus, it is recommended to store films in a cool and dark environment [42]. There is a possibility to use cyanine-based infrared dyes (like IR-783 and IR-806) in gelatin matrix dosimeters, but the absorbance can decay up to 30–60% over 176 days following an exponential decay function [43]. In this study, the feasibility of PVA-based films supplemented with AgNPs and with various organic dyes, including methyl red (MR) and toluene blue (TBO), to serve as radiation detectors/radiation indicators was assessed. These films were investigated in the dose range between 0 Gy and 10 Gy over 180 days.

## 2. Results and Discussion

The feasibility of silver-enriched PVA films with and without azo dye additives to serve as radiation exposure indicators and dosimeters was investigated, analyzing the optical properties of the experimental films irradiated with doses from the interval of 0–10 Gy. Because radiation detectors containing Ag nanoparticles are very photosensitive and experience an aging effect [44,45], the performed analysis included an assessment of time-dependent changes in the optical parameters of the experimental films during the post-irradiation period ranging up to 180 days.

To investigate the radiosensitivity of colored AgPVAGly films and evaluate the stability of their film properties over the post-irradiation period, the performance of the AgPVAGly films without dye additives was assessed in the first step.

### 2.1. AgPVAGly Films

Already, the first visual inspection of the as-prepared AgPVAGly films showed a light yellowish color of the dried films. This coloration of the films was attributed to the possible formation of silver seeds from silver nitrate during the drying period and their aggregation to silver nanoparticles, which was stimulated via interactions between the silver ions and PVA due to the decreasing amount of water in the samples [40]. The film color became more intense after the irradiation of the films with 6 MeV X-ray photons within the dose interval from 0 Gy to 10 Gy, because even relatively low photon irradiation doses contribute to the formation of silver nanoparticles via radiolysis processes [35,40,42,46]. Moreover, dose-dependent variations in the film color were assessed during the post-irradiation period (Table 1), indicating some applicability limits of these films as exposure sensors.

A more detailed investigation of the irradiated AgPVAGly films was performed, analyzing the UV-Vis absorbance spectra. A low-intensity broad local surface plasmon resonance peak (LSPR) was observed at a 455 nm wavelength for the as-prepared films, indicating the formation of Ag seeds and small amounts of differently sized AgNPs, already during the drying process of the films. The irradiation of films with relatively low doses (0–10 Gy range) led to a more intense synthesis of nanoparticles of a dominating spherical shape, which correlated well with a slight shift in a better-pronounced LSPR peak towards 445 nm. The intensity of the LSPR peak of the films irradiated with 10 Gy was twice as high as that of the initial unirradiated films. The UV-Vis absorbance spectra fragments of the irradiated AgPVAGly films with the observed LSPR peaks are provided in Table 2.

It should be noted that the first evaluation of the irradiated films was performed on the same day after irradiation, since exposure indicators are thought to react to radiation immediately, and this reaction can be followed by the color changes in the irradiated films. However reliable dosimetric information from irradiated films can be extracted a few days after irradiation, because low-dose irradiation also contributes to some rearrangements in the polymeric structures of the films that need some time to achieve thermal equilibrium.

Taking this into account, the irradiated samples were stored in a dark place at 20 °C for some time and were evaluated after 1 week (7 days), 4 weeks (30 days), 6 weeks (45 days), and 6 months (180 days) post-exposure. Fragments of the UV-Vis spectra with the LSPRs of differently irradiated films obtained at 7 days and 45 days after the irradiation are provided in Table 2, respectively.

**Figure 1:**
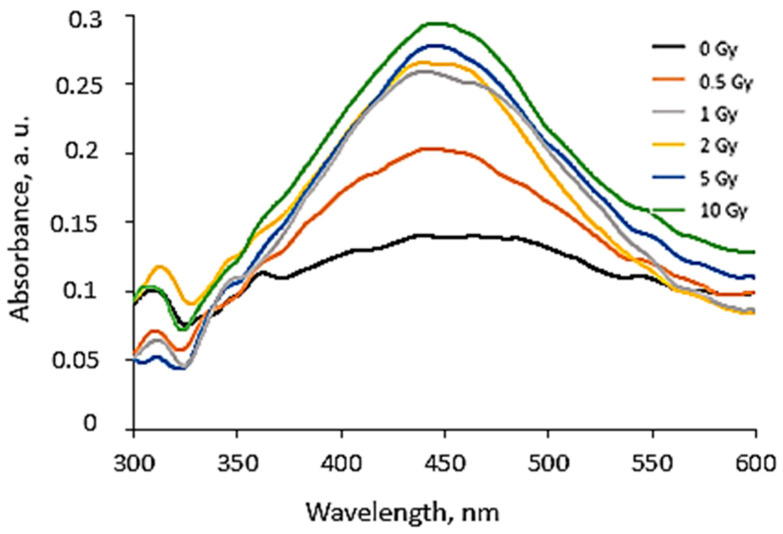
UV-Vis absorbance spectra of the irradiated AgPVAGly films, evaluated 180 days after irradiation.

The increasing dose-related tendency of the LSPR peak intensity was observed with a prolonged time of irradiated film storage due to the continuous self-assembly processes of AgNPs within the polymer network. However, already after 3 months and especially after 6 months, the irradiation aging effect of the films was observed, contributing to the significant color changes in the films (see Table 1). Due to the loss of water after a prolonged time after exposure, the agglomeration of the AgNPs started to occur, leading to a time-dependent steady decrease in the LSPR peak intensity, which dropped down to the absorbance level of 0.3 a.u. (Figure 1).

Taking into account changes in the optical properties of the irradiated AgPVAGly films due to the prolonged time between their exposure and evaluation, the dose sensitivity and stability of the dose-related information were investigated within 1–45 days. For this reason, the LSPR peak intensity of the films irradiated with different doses was assessed after a corresponding period. The dose sensitivity was calculated as a slope of the dose dependency of the irradiated film LSPR peak’s intensity.

**Figure 2:**
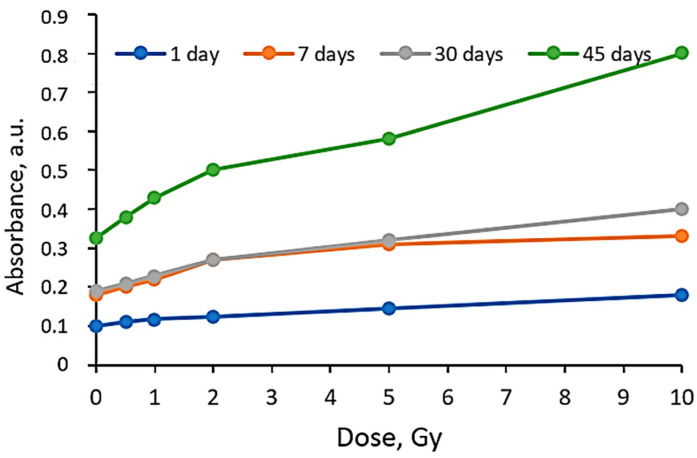
Sensitivity of AgPVAGly films irradiated with different doses and evaluated after a certain post-irradiation period.

It was found that the dose sensitivity of the AgPVAGly films was rather low, indicating an increasing tendency with time after irradiation (Figure 2). The films were more sensitive to doses up to 2 Gy, indicating the potential of silver-enriched PVA as a low dose detector.

**Figure 3:**
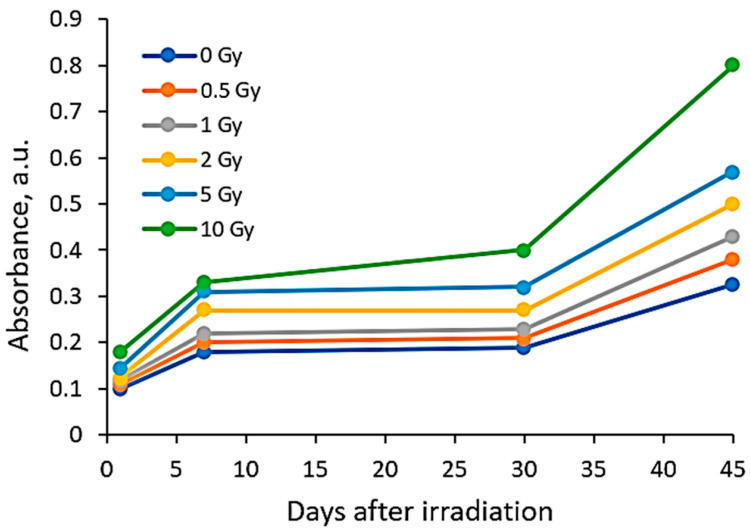
Post-irradiation time-dependent LSPR peak intensity variations in the films irradiated with different doses.

The performed analysis revealed that the dose sensitivity, evaluated just after the film irradiation, was almost negligible within the whole period of investigation. The highest sensitivity of 0.086 Gy^−1^ was estimated for the irradiated films stored for 45 days before evaluation. Almost the same sensitivity of ~0.051 Gy^−1^ for the doses up to 2 Gy was found in the evaluated films after 7 days and 30 days. These results supported our findings, obtained by analyzing the stability of the dose-related parameters during the time (Figure 3).

It was found that, to obtain reliable exposure dose-related results, the AgPVAGly films should be evaluated within a period between 7 days and 30 days. During this period, the dose sensitivity of differently irradiated films remained stable. However, after a longer period, a deterioration in dose sensitivity was observed, indicating time-related limitations for the extraction of dose-related information from the exposed (irradiated) films.

### 2.2. Silver-Enriched PVA Films with Dye Additives

To assess the performances of the silver-enriched PVA films with azo dye additives as radiation dosimeters/exposure indicators, the experimental films were investigated after their irradiation with certain doses following the same procedure as that discussed in the previous chapter.

In contrast to the silver-enriched PVA films that gained color due to the formation of AgNPs during irradiation, azo dyes as adducts to the AgPVAGly films caused them to lose their color due to the reaction of hydroxyl radicals (OH^●^) produced as water radiolysis products with the azo (N=N) group [47,48].

In this article, we investigated PVA-based films containing two types of azo dyes:

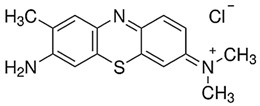

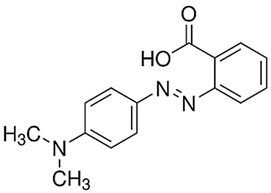
Toluidine Blue OMethyl red

**Figure 4:**
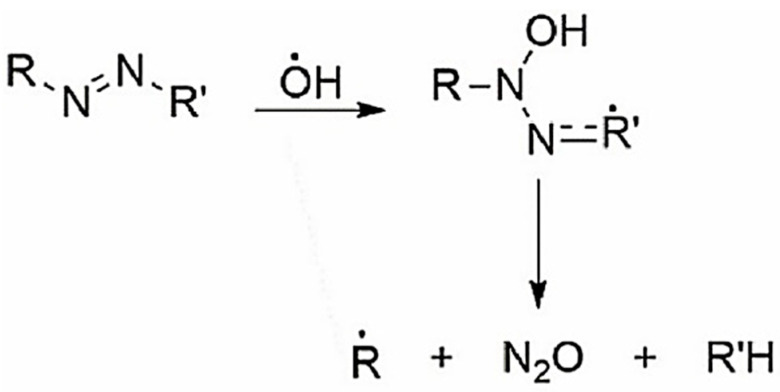
Destruction of -N=N- bond in azo dye by attack of OH^●^ radical.

It is known [42,47,49] that the double bond in the azo group (N=N) is relatively weak and there is a high probability that it can be easily broken via the interaction with OH^●^ (Figure 4).

The addition of an OH^●^ to a double bond damages the intramolecular conjugation system, making it shorter. In turn, the unpaired electron delocalizes over the remaining conjugated bonds (including the aromatic unit). As a result, conformational stresses arise in the OH adduct due to the mismatch between the electronic configurations of the OH adduct and the initial unit. Relaxation is possible in a rigid dye molecule [42,47,49] due to the cleavage of C–N, C–O, and C–C bonds. These processes lead to a reduction in or elimination of color.

It should be noted that the solvated electrons e_aq_^−^ and H^●^ also play an important role in the reduction in intense color via the destruction of the -N=N- bond (color-giving center) of azo dye [47,49]. The decolorization process of azo dyes containing PVA films and AgNPs-enriched PVA films was thoroughly discussed in our previous paper [38]. The recent investigation was focused on the evaluation of the optical properties of AgPVAGly films that contain azo dye additives and their post-irradiation time-dependent variations.

### 2.3. AgPVAGlyTBO Films

The variations in the optical properties (fragments of the absorbance spectra of the irradiated AgPVAGlyTBO films) evaluated at different time points of the post-irradiation period are provided in Table 3. The color variations during the post-irradiation period of the same films are shown in Table 4.

A visual inspection of the differently irradiated AgPVAGlyTBO films led to the conclusion that the intensity of color decreased with an increasing irradiation dose. Film color bleaching after a prolonged irradiation time was also observed. These findings fit well with the results retrieved by an analysis of the UV-Vis absorbance spectra. A shoulder with a small peak at 482 nm corresponding to AgNPs’ formation (LSPR peak) was seen in all investigated spectra. This peak modestly grew during a certain period after irradiation, thus following a tendency which was observed for the AgPVAGly films without additives. On the contrary, a color-related peak at 624 nm, corresponding to the presence of Toluidine Blue O dye (Chempur Piekary Śląskie, Poland) in the films, dominated and decreased with an increased irradiation dose. Decolorization of the films was also observed after irradiation, indicating the radiation-induced destruction of azo groups.

**Figure 5:**
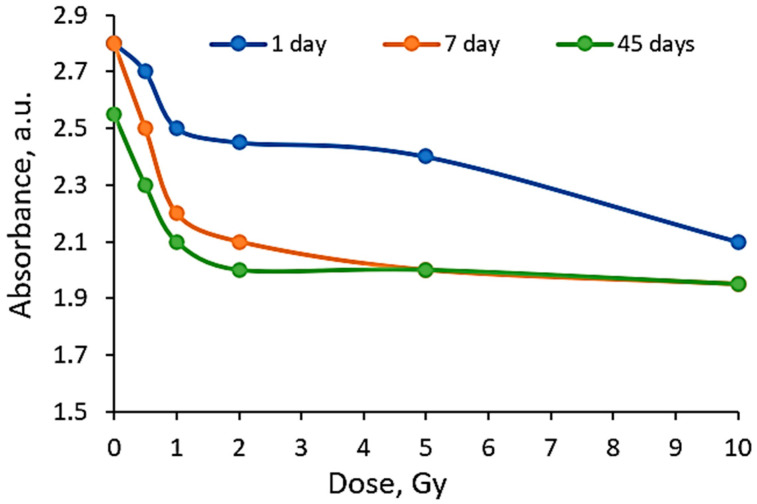
Sensitivity of AgPVAGlyTBO films irradiated with different doses and evaluated after a certain post-irradiation period.

In general, a dose sensitivity enhancement effect was achieved in the AgNPs containing PVA films with Toluidine Blue O dye adducts. It was found (Figure 5) that these films were very sensitive to irradiation in the low dose range (up to 2 Gy). The estimated average sensitivity of the films varied between 0.3 Gy^−1^ and 0.61 Gy^−1^ and was significantly higher as compared with the sensitivity of 0.057 Gy^−1^ estimated in our previous paper [38] for PVA-TBO films without AgNPs. The film response to doses of >2 Gy were small, and only very small color changes were observed. The observed color bleaching of the films was evaluated using the formula provided in [48].

(1) $$Color\;bleaching=\frac{A_{0}-A}{A_{0}}\times 100\%,$$

where *A*_0_ is the film optical absorption intensity at a zero dose and *A* is the film optical absorption after irradiation with a certain dose. Due to the same dose rate during each irradiation procedure, the total bleaching of the AgPVAGlyTBO films irradiated to 10 Gy doses was 28.6%, but already after 2 Gy irradiation, film bleaching by 21.4% was achieved, indicating a higher irradiation effectiveness/film responsiveness during a shorter irradiation time. The bleaching effect was almost twice as intense as for the PVA-TBO film without AgNPs.

It is important to admit that, as in the case of AgPVAGly films discussed before, reliable dosimetric parameters were obtained 7 days after irradiation.

### 2.4. AgPVAGlyMR Films

It should be noted that Methyl Red was not fully dissolved in the PVA gel solution, which implied the occurrence of small dye particulates in the dried AgPVAGlyMR films, thus indicating that dose-related tendencies (only!) for the optical properties’ changes can be evaluated properly. The performed visual inspection of these films indicated a color degradation tendency with an increased irradiation dose and also with post-irradiation time (Table 5).

Because the absorbance peak of Methylene Red (~555 nm) is closely located to the AgNPs-related LSPR peak at 455 nm, as was estimated in the previous chapter, the UV-Vis absorbance peak of the irradiated AgPVAGlyMR films is a result of two overlapping peaks and represents two competing processes: the growth of the LSPR peak intensity due to the radiation-induced formation of AgNPs and a decreasing intensity of the MR-related peak due to the radiation-induced decolorization of the film. Taking into account that the reduction in MR color dominated over the AgNPs’ formation process, as was shown in our previous paper [38], the resulting absorbance peak of the irradiated AgPVAGlyMR films was found at 482 nm and was slightly shifted towards longer wavelengths (488 nm) after the long post-irradiation period (45 days). Dose-dependent variations in the UV-Vis absorbance peak during the post-irradiation period are provided in Table 6.

**Figure 6:**
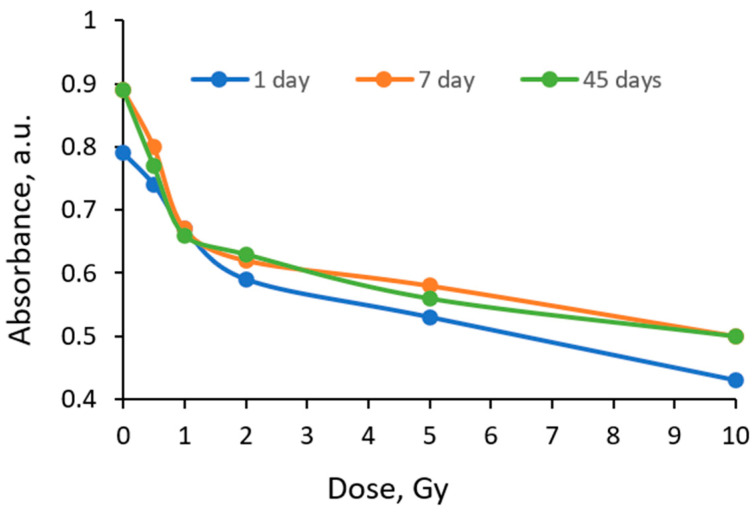
Dose sensitivity of AgPVAGlyMR films irradiated with different doses and evaluated after a certain post irradiation period.

The evaluation of the AgPVAGlyMR film dose response revealed that the formation of AgNPs due to irradiation increased the overall intensity of the UV-Vis absorbance peak of the irradiated film (Figure 6), however, a descending tendency of this peak was observed with an increased radiation dose. Similar tendencies of optical properties’ variations in irradiated polymer films containing azo dyes have also observed been by other authors [47,50,51], however, in almost all cases, films/gels were evaluated within a short period after irradiation. Also, it should be admitted that there are only few articles discussing NPs-enriched films containing MR dye as the adduct.

Almost no significant changes in the optical properties were observed for the films evaluated between 7 and 45 days after exposure. However, the overall dose sensitivity of the AgPVAGlyMR films irradiated with a dose of <2 Gy was higher (0.24 Gy^−1^), as compared to the dose sensitivity of 0.11 Gy^−1^ which was found for PVA-Methyl Red dye films in our previous paper [38]. Color bleaching by 26.7% was achieved after film irradiation to a 1 Gy dose, and the total bleaching was 38.9%. The later information regarding variations in the dose-dependent optical properties of the irradiated films was extracted 7 days after irradiation.

## 3. Conclusions

Thin (~200 µm) and flexible silver-containing PVA-based gel films with and without azo dye additives were produced. It was shown that the reliable dose-related parameters were obtained one week (7 days) after irradiation and remained almost stable up to 30–45 days, indicating variations on the level of 1%. All investigated films were more sensitive to low-dose (up to 2 Gy) photon irradiation.

The irradiated AgPVAGlyTBO films demonstrated the highest dose response (0.61 Gy^−1^) of UV-Vis absorbance peak intensity among all investigated films, which was much higher than the dose sensitivity (0.057 Gy^−1^) evaluated for just PVA-TBO films. A large enhancement of dose sensitivity was possible, because no overlapping of the LSPR peak corresponding to the formation of AgNPs at 482 nm and the UV-Vis peak of toluidine blue at 624 nm was found, thus indicating a prevailing reduction in the UV-Vis absorbance peak of the whole AgPVAGlyTBO film with the increased dose.

The AgPVAGlyMR films were less sensitive than the AgPVAGlyTBO films, but much more sensitive (0.24 Gy^−1^) as compared to the PVA-MR films (0.11 Gy^−1^) in the dose range up to 2 Gy. The lower sensitivity of AgPVAGlyMR compared to the silver-enriched PVA films containing TBO dye was due to the fact that UV-Vis peaks representing two competing processes during irradiation were located close to each other and a reduction in the MR-related UV-Vis absorbance peak was inhibited by the increase in the LSPR peak corresponding to the formation of AgNPs. However, the color bleaching effect was higher for the AgPVAGlyMR films (26.7%) as compared to 21.4% for the AgPVAGlyTBO films irradiated to low (up to 2 Gy) doses.

The performed investigation revealed the applicability of silver-enriched PVA-based films with azo dye adducts as potential low-dose radiation detectors and exposure indicators.

## 4. Materials and Methods

PVA thin films enriched with silver nanoparticles with and without dye additives were fabricated for the dose assessment. The chemical compositions of the prepared thin films are provided in Table 7.

The materials used for this investigation without any further modifications were directly purchased from the corresponding companies: polyvinyl alcohol (C_2_H_8_O_3_, M_w_~125.000 MOWIOL^®^ 20-98 MW; Sigma-Aldrich Chemie GmbH, Regensburg, Germany), silver nitrate (AgNO_3_, purity > 99.9% Molar Chemicals Kft, Halásztelek, Árpád, Hungary, glycerol (C_3_H_8_O_3_, Chempur Piekary Śląskie, Poland), ethanol (C_2_H_5_OH, purity ~96%, Euro-Chemicals GmbH, Nordhorn, Germany), analytic-grade water for laboratory use, Chempur Piekary Śląskie, Poland, toluidine blue O (C_15_H_16_ClN_3_S, Chempur Piekary Śląskie, Poland), and Methyl red (C_15_H_15_N_3_O_2_, Chempur Piekary Śląskie, Poland).

### 4.1. Film Fabrication

**Figure 7:**
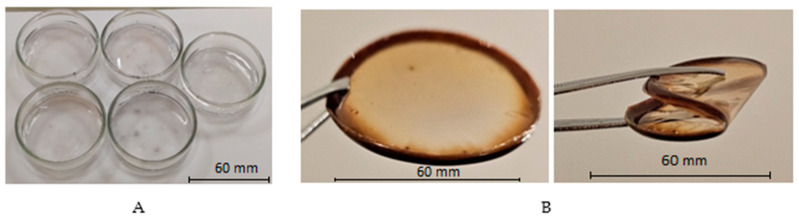
Fabrication of films: (**A**)—as-prepared AgPVAGly solutions poured into Petri Dishes and (**B**)—dried flexible AgPVAGly thin films.

In the first step, a certain amount of PVA was dissolved in distilled water under continuous stirring using a Steinberg Systems (Berlin, Germany) SBS-MR-1600/1T PRO magnetic stirrer at a constant 150 RPM for 40 min and maintained at a 70 °C temperature. The produced 10% PVA water solution was cooled down to room temperature and 6 g (0.86%) of AgNO_3_ was added drop by drop, continuing stirring for a couple of minutes until the complete dissolution of salt. To increase the PVA solubility in water and prevent the agglomeration of silver nanoparticles, 3 g (4.28%) of glycerol and 0.35 g (0.50%) of ethanol were added to the solution instead of water. Unlike glycerol, admixed ethanol was responsible for the stabilization of Ag nanoparticles in the PVA matrix [40]. The prepared colorless polymer gel solution (Figure 7A) was cast into standard Petri dishes and dried for 72 h in the dark at 20 °C until ~200 µm thick, flexible, slightly yellowish films were formed (Figure 7B). A reduction in water amount during the entire drying process was responsible for the reduced adhesion force between the gel layer and the surface of the Petri dish, thus making the removal of films from Petri dishes relatively simple.

Colored films were produced following the same fabrication procedure, but adding a certain amount of the dyes (TBO and MR) dissolved in ethanol in the final step.

Over the whole period of investigation, the experimental films were stored in a dark place at room temperature, except during the irradiation and evaluation sections.

### 4.2. Irradiation of Experimental Films

The prepared films were irradiated in a linear accelerator Varian TrueBeam 2.1 STx using 6 MeV photons delivered to the target with a dose rate of 600 MU/min (4.61 Gy/min) at the Oncology Institute of the Lithuanian University of Health Sciences. Doses in the range of 0–10 Gy were applied for irradiation. Thin films were placed between two 1 cm thick PMMA plates and the whole irradiation construction consisted of 5 plates with the same thickness above the film and 5 plates below the film. The source-to-surface distance (SSD) of 100 cm was kept in all experiments, as well as a Gantry rotation of 180° and a field size of 30 × 30 cm^2^. Doses from the range of 0–10 Gy were delivered to the films and checked with a 0.6 cm^3^ sensitive volume PTW (Freiburg, Germany) Farmer TM30013 ionization chamber.

### 4.3. Characterization of Experimental Films

Radiation-induced changes in the irradiated PVA films enriched with silver nanoparticles with and without dye additives were characterized using UV-Vis spectra obtained using the spectrophotometer Ocean Optics with USB 4000 (Ocean Optics, Inc., Dunedin, FL, USA). Light source—HL-2000-LL by Ocean Insight—long lifetime tungsten halogen with wavelength range of 360 nm–2.4 µm and power of 4.75 W. Variations in the optical characteristics of the films were analyzed in the range between 350 and 900 nm using “Ocean View” spectroscopy software (version 1.6.7 by Ocean Optics).

## Figures and Tables

**Table 1:** Irradiation dose-dependent variations in AgPVAGly film color changes stored in the dark at 20 °C during post-irradiation period.

Dose, Gy	0	0.5	1	2	5	10
On the same day of irradiation	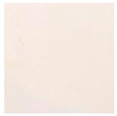	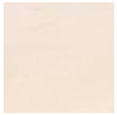	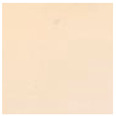	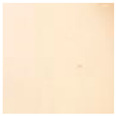	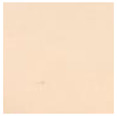	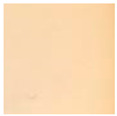
7 days after irradiation	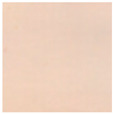	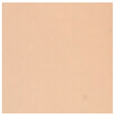	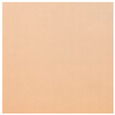	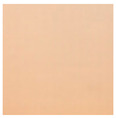	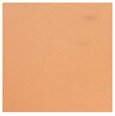	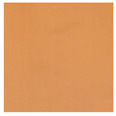
30 days after irradiation	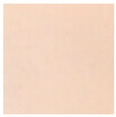	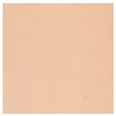	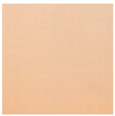	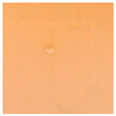	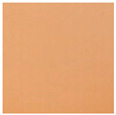	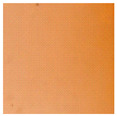
45 days after irradiation	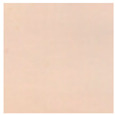	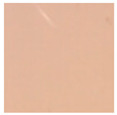	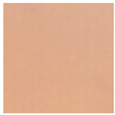	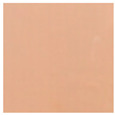	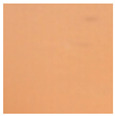	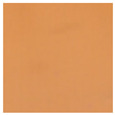
90 days after irradiation	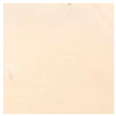	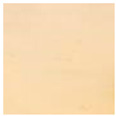	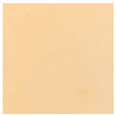	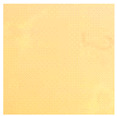	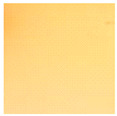	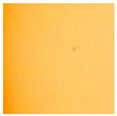
180 days after irradiation	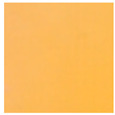	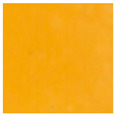	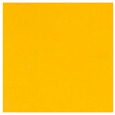	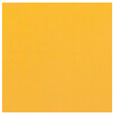	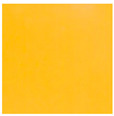	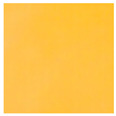

**Table 2:** Fragments of UV-Vis absorbance spectra of irradiated AgPVAGly films.

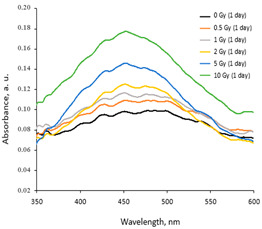	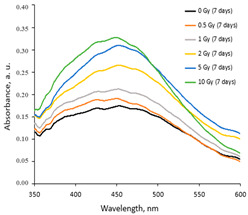	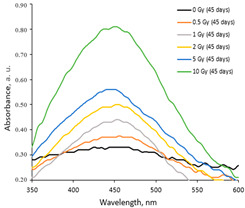
On the same day of irradiation	After 7 days post irradiation	After 45 days post irradiation
*Different scales on Absorbance axis for AgPVAGly films*		

**Table 3:** Fragments of UV-Vis absorbance spectra of irradiated AgPVAGlyTBO films.

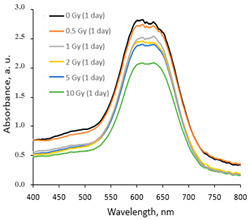	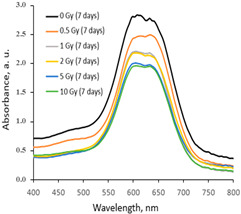	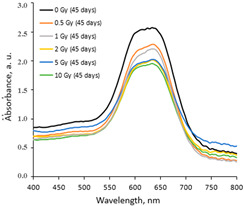
Within the same day of irradiation	After 7 days post irradiation	After 45 days post irradiation

**Table 4:** Color variations during post irradiation period of the AgPVAGlyTBO films irradiated with different doses.

Dose, Gy	0	0.5	1	2	5	10
On the same day of irradiation	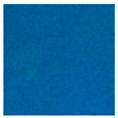	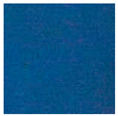	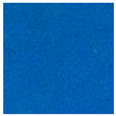	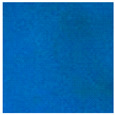	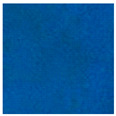	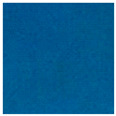
7 days after irradiation	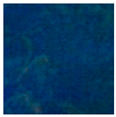	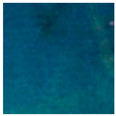	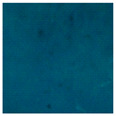	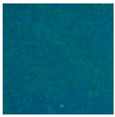	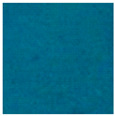	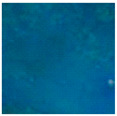
30 days after irradiation	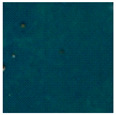	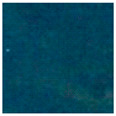	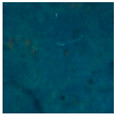	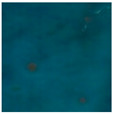	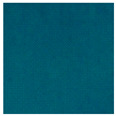	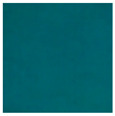
180 days after irradiation	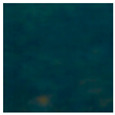	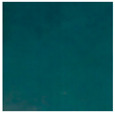	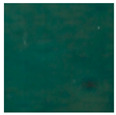	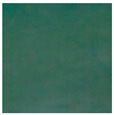	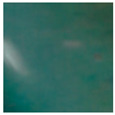	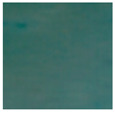

**Table 5:** Color variations during post-irradiation period of the AgPVAGlyMR films irradiated with different doses.

Dose, Gy	0	0.5	1	2	5	10
On the same day of irradiation	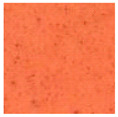	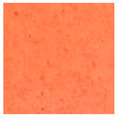	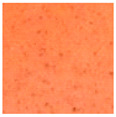	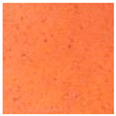	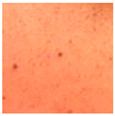	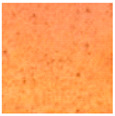
7 days after irradiation	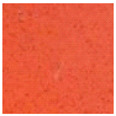	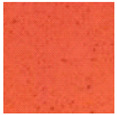	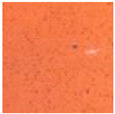	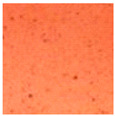	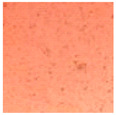	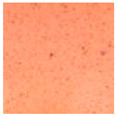
30 days after irradiation	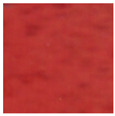	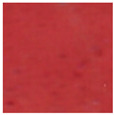	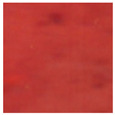	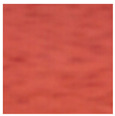	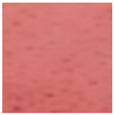	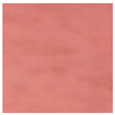
180 days after irradiation	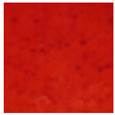	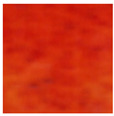	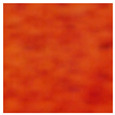	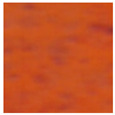	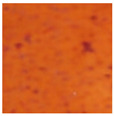	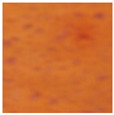

**Table 6:** Fragments of UV-Vis absorbance spectra of irradiated AgPVAGlyMR films.

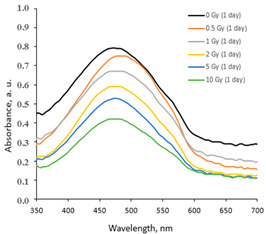	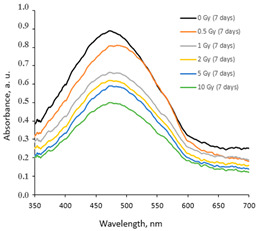	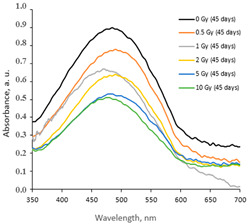
On the same day of irradiation	After 7 days post irradiation	After 45 days post irradiation

**Table 7:** Chemical compositions of the prepared colored PVA films enriched with Ag nanoparticles.

Material	Chemical Formula	PVA-Ag-Gly, %	PVA-Ag-Gly-TBO, %	PVA-Ag-Gly-MR, %
Polivynyl alcohol, (PVA)	C_2_H_8_O_3_	8.62	8.58	8.58
Silver nitrate	AgNO_3_	0.86	0.86	0.86
Glycerol (Gly)	C_3_H_8_O_3_	4.31	4.28	4.28
Ethanol (Etha)	C_2_H_5_OH		0.5	0.5
Toluidine blue O (TBO)	C_15_H_16_ClN_3_S		0.01	
Methyl red (MR)	C_15_H_15_N_3_O_2_			0.01
Distilled water	H_2_O	86.21	85.77	85.77

## Data Availability

The data presented in this study are openly available in article.
